# Hit series selection in noisy HTS data: clustering techniques, statistical tests and data visualisations

**DOI:** 10.1186/1758-2946-6-S1-P27

**Published:** 2014-03-11

**Authors:** Christoph Müller, Daniel Ormsby, Isabella Feierberg, Ola Engkvist, Christian Tyrchan, Michael J Hartshorn

**Affiliations:** 1 Dotmatics Ltd, Windhill, Bishop’s Stortford, CM23 2ND, UK; 2 AstraZeneca AB, Peppardsleden 1, Mölndal, 43183, Sweden

## 

High throughput screening (HTS) is one of the most prominent techniques used in the beginning stages of a drug discovery programme to identify those few hit compounds that can be used as starting points in subsequent studies [1,2]. However, an HTS experiment often entails a very data-intensive and challenging hit prioritization process that yields the mentioned hit compounds. The workflow described in this study aims to make this decision-making process easier by combining the structural and biological information of compounds used in an HTS. In particular, the workflow combines various clustering and nearest neighbourhood schemes with a non-parametric statistical test in order to prioritize those groupings of compounds that are likely of being relevant to the biological target of interest [3].

The novel workflow was evaluated under various aspects in a retrospective study using publicly available quantitative HTS (qHTS) datasets [4]. One of the main benchmarking aspects in this study was the ability to correctly identify as many true active compounds as possible. Therefore different chemical descriptors and clustering schemes were tested in combination with the statistic to measure their classification performance.

The workflow was integrated into Dotmatics’ *Vortex*, a platform for analysing chemical information using chemoinformatics methods and data visualisations tools [5]. This integration enables researchers to easily extend their current HTS workflow in order to discover new hit series and reveal hidden relationships between compounds, scaffolds and clusters.
